# Establishment and characterization of a non-gestational choriocarcinoma patient-derived xenograft model

**DOI:** 10.1186/s12885-023-11626-3

**Published:** 2023-11-13

**Authors:** Yukari Oda, Kaoru Niimi, Kosuke Yoshida, Satoshi Tamauchi, Akira Yokoi, Yuko Yasui, Yuki Nishiko, Mayu Shibata, Yusuke Shimizu, Masato Yoshihara, Yoshiki Ikeda, Nobuhisa Yoshikawa, Kimihiro Nishino, Eiko Yamamoto, Hiroaki Kajiyama

**Affiliations:** 1 Department of Obstetrics and Gynaecology, Nagoya University Graduate School of Medicine, Tsuruma- cho 65, Showa-ku, Nagoya, 466-8550 Japan; 2 Institute for Advanced Research, Nagoya University, Tsuruma-cho 65, Showa-ku, Nagoya, Japan; 3 Department of Healthcare Administration, Nagoya University Graduate School of Medicine, Tsuruma- cho 65, Showa-ku, Nagoya, Japan

**Keywords:** Germ cell tumour, Non-gestational choriocarcinoma, Ovarian cancer, Patient-derived xenograft model, RNA sequencing

## Abstract

**Background:**

Non-gestational choriocarcinoma (NGC) is a rare subtype of malignant germ cell tumour and there is no consensus on its treatment. The lack of suitable preclinical models for NGC is a challenge in drug discovery research. Patient-derived xenograft (PDX) models recapitulate the tumour microenvironment of the original cancer tissue. Therefore, they have received considerable attention for studies on rare cancer. Here, we aimed to establish a PDX model from a patient with recurrent NGC.

**Methods:**

Fresh NGC tumour tissue was immediately transplanted into a severely immune-deficient mouse (NOD.Cg*-Prkdc*^*scid*^*1l2rg*^tm1Wjl^/SzJ) and maintained for more than three in vivo passages. Subsequently, we evaluated the molecular characteristics of the PDX model using immunohistochemistry, polymerase chain reaction, and RNA sequencing. Moreover, the PDX tumours were transplanted into BALB/c nude mice, and we evaluated their sensitivity for cisplatin and methotrexate.

**Results:**

The PDX tumour maintained the morphological features of NGC. Moreover, Immunohistochemistry revealed that the human chorionic gonadotropin, cytokeratin 7, and EpCAM expression levels were similar to those in the primary tumour. Furthermore, serum human chorionic gonadotropin levels were elevated in both the primary tumour and the PDX models. Additionally, using PCR analysis with species-specific primers, we confirmed that the PDX tumour contained human genes and was derived from human tissue. Moreover, the gene expression profile of the NGC was compared with that of epithelial ovarian cancer samples and cell lines, and 568 dysregulated genes in the NGC were extracted. The expression of the dysregulated genes in PDX was significantly correlated with that in the primary tumour (R^2^ = 0.873, *P* < 0.001). Finally, we demonstrated that the PDX tumour was sensitive to cisplatin and methotrexate; therefore, its clinical response to the agents was similar to that of the primary tumour.

**Conclusions:**

We successfully established a PDX model of NGC, to the best of our knowledge, for the first time. The established PDX retained the molecular and transcriptome characteristics of the primary tumour and can be used to predict drug effects. It may facilitate further research and the development of novel therapeutic agents for NGC.

**Supplementary Information:**

The online version contains supplementary material available at 10.1186/s12885-023-11626-3.

## Background

Choriocarcinoma is a malignant tumour that forms when trophoblastic cells, a part of the placenta, proliferate abnormally and become tumour-like [1]. Choriocarcinoma is classified into two subtypes, gestational and non-gestational, depending on its presumed origin. Gestational choriocarcinoma (GC) can arise from any gestation, including normal pregnancy, abortion, and molar pregnancies; therefore, GC is classified as gestational trophoblastic neoplasia (GTN). Conversely, non-gestational choriocarcinoma (NGC) is one of the malignant germ cell tumours (MGCTs), which originates from primordial germ cells. Hence, NGC usually arises in the female genital tract and rarely in other extra-genital organs, such as the retroperitoneum or mediastinum [2]. The incidence of ovarian NGC is less than 0.6% among all ovarian neoplasms [3, 4]. Although GC and NGC have similar histological features; their backgrounds are different. The clinical symptoms of NGC are non-specific, such as lower abdominal pain and genital bleeding, Moreover, NGC is often characterized by invasion into adjacent organs and extensive metastasis to distant organs, particularly the brain and lungs [5, 6]. A previous study reported that about a half of the patients with ovarian NGC are advanced-stage cases (Stage I, II, III, and IV: 41.2%, 5.9%, 5.9%, and 47.0%, respectively; the 2013 FIGO staging for ovarian cancer) [1].

Human chorionic gonadotropin (hCG), which is secreted by trophoblasts, is a useful biomarker for diagnosis and monitoring the treatment response. In addition to pathological findings, a history of sexual intercourse and pregnancy is critical information that could facilitate distinguishing between GC and NGC. The standard treatment for NGC has not been established; however, surgical removal of the tumour and systemic chemotherapy are effective treatment options [7]. Particularly, MGCTs are expected to be highly sensitive to chemotherapy. According to the National Comprehensive Cancer Network (NCCN) and European Society for Medical Oncology (ESMO) guidelines, the BEP therapy (Bleomycin, Etoposide, and Cisplatin) is the most commonly used regimen for MGCT. In addition, the MEA therapy (methotrexate, etoposide, and dactinomycin) can be an alternative treatment for GTN [2]. Reportedly, the prognosis of ovarian NGC is worse than that of GC [2, 8, 9]. According to a previous study, the 3-year cumulative overall survival rate was 100% for patients with Stage I, II, and III cancer; however, it decreased to 25% for patients at Stage IV [2]. To improve the prognosis of NGC, further drug discovery research is greatly required. However, conducting large-scale clinical studies is challenging. Furthermore, there is no suitable preclinical model for NGC due to low incidence.

Patient-derived xenograft (PDX) models are created by transplanting the patient’s tumour tissue into immune-deficient mice. PDX tumours retain the properties of primary patient tumours, such as glandular, vascular, and stromal structures; cellular complexity; and cytogenetic features [10–12]. In addition, multiple studies have demonstrated that PDX tumours have molecular expression profiles similar to the primary tumours even after several passages [13–15]. The response of PDX tumours to anticancer drugs is also consistent with that of patient tumours [16]. Therefore, the PDX model is useful for cancer biology studies and preclinical research on novel therapeutic agents, including molecular-targeted and conventional anticancer drugs, for precision cancer medicine [17, 18].

In the present study, a PDX model of NGC was established successfully for the first time, to the best of our knowledge. Additionally, the histological, molecular, and pharmacological characteristics of the PDX model were investigated. The PDX model could facilitate the elucidation of the molecular background of NGC and the development of novel therapeutic strategies.

## Methods

### Patients

One patient with a gynaecological malignancy was included. To establish the PDX models, a residual tumour was obtained from the left ovary during a fourth surgical procedure, and clinical data of the patient were collected. The study protocol was approved by the ethics committee of the Nagoya University Graduate School of Medicine in Japan (approval number 2015 − 0237), and written informed consent was obtained from the patient. We also confirmed that the consent to publish was obtained from the study participant.

### Animal experiments

Four-week-old female NSG (NOD.Cg*-Prkdc*^*scid*^*Il2rg*^*tm1Wjl*^/SzJ) and BALB/c nude mice were purchased from Oriental Bio Service, Inc. (Kyoto, Japan). The mice were maintained in a pathogen-free, temperature-controlled environment with a 12 h light/dark cycle, and NSG mice were fed autoclaved water and irradiated food. The mice were carefully monitored, and tumour volumes were calculated using the following formula: volume = length × width × width × 1/2. NSG mice were used for the PDX model establishment. BALB/c nude mice were used in experiments to observe how the established PDX model responded to the drugs. The humane endpoint criteria required euthanasia if the tumour length diameter was > 2 cm, weight loss in a few days was > 20%, or self-mutilation occurred. All animal experiments were approved by the Animal Care and Use Committee of the Nagoya University Graduate School of Medicine (Nagoya, Japan).

To establish the PDX model, a residual tumour was obtained from the left ovary during a fourth surgical procedure. The tumour was washed with PBS and sectioned into approximately 8 mm^3^ pieces. Thereafter, six tumour sections were subcutaneously transplanted into NSG mice under an anaesthetic combination of 0.75 mg/kg of medetomidine (Kyoritsu Seiyaku, Tokyo, Japan), 4.0 mg/kg of midazolam (Sandoz, Tokyo, Japan), and 5.0 mg/kg of butorphanol (Meiji Seika Pharma, Kyoto, Japan). When the tumour length diameter reached approximately 2 cm, the tumours were harvested and passed. The generation harbouring the patient-derived material was termed P1, with subsequent generations numbered consecutively (P2, P3, P4, etc.). The PDX model was considered established after the P3 generation, and the tumours were cryopreserved using liquid nitrogen in CELLBANKER (Takara, Shiga, Japan).

For the drug sensitivity assay, 10 BALB/c nude mice were subcutaneously transplanted with PDX tumours (P6 generation). From the viewpoint of animal welfare, the number of experimental mice was set to the minimum effective number. They were then randomly divided into two groups when the tumour volume reached approximately 100 mm^3^. The mice received intraperitoneal administrations of either cisplatin (CDDP, 2 mg/kg body weight, Nichi-Iko, Toyama, Japan) or PBS four times every 2 days. Similarly, for the second assay, 10 mice were subcutaneously transplanted with PDX tumours (P7 generation) and randomly assigned to two groups. Thereafter, they received intraperitoneal administrations of either methotrexate (MTX, 50 mg/kg body weight, Pfizer, New York, NY, USA) or PBS four times every 3 days. All mice were sacrificed when the tumour volume was > 1500 mm^3^.

### Immunohistochemistry

The PDX tumour tissues were fixed in 4% paraformaldehyde for 12–16 h and embedded in paraffin. Formalin-fixed and paraffin-embedded tissues were sliced at a thickness of 4 μm using a microtome. Sections were deparaffinized and rehydrated in xylene and a gradient series of ethanol. For antigen retrieval, the sections were heated in Tris/EDTA buffer (pH 9.0) for 20 min at 90 °C (for cytokeratin 7 (CK7) and EpCAM) in a microwave. Antigen retrieval was not performed for hCG. Thereafter, to block endogenous peroxidase, the sections were incubated with 0.3% H_2_O_2_ in methanol for 10 min (for CK7 and EpCAM) or 0.03% H_2_O_2_ in methanol for 10 min (for hCG). Subsequently, the sections were blocked with appropriate serum using the SAB‑PO (R) kit (Nichirei, Tokyo, Japan), according to the manufacturer’s protocol (for CK7 and EpCAM), or 1% BSA in PBS (for hCG). Thereafter, the sections were incubated with the following primary antibodies at 4 °C for 12–16 h: anti-hCG Ab (1:1; N1534, Dako, Glostrup, Denmark), anti-CK7 Ab (1:8000; ab181598, Abcam, Cambridge, MA, USA), and anti-EpCAM Ab (1:16000; ab213500, Abcam). The CK7- and EpCAM-treated sections were incubated with appropriate secondary antibody for 10 min, followed by peroxidase-labelled streptavidin for 5 min using the SAB‑PO (R) kit; the hCG-treated sections were incubated with the secondary antibody conjugated to horseradish peroxidase-labelled polymer (EnVision + anti-Rabbit; Dako) for 60 min. After incubation, the sections were stained with the DAB working reagent for 4–15 min. Finally, the sections were counterstained with haematoxylin, dehydrated, and mounted. All images were acquired using a Zeiss Axio Imager A1 microscope (Carl Zeiss Meditec AG, Jena, Germany).

### Determining the plasma hCG level

Blood samples were collected by cardiac puncture in the mice at the time of their sacrifice and centrifuged at 2,000 ×*g* for 10 min at 15–25 °C. Plasma samples were analysed using enzyme immunoassays (CLEIA) with α-hCG and β-hCG-CTP monoclonal antibodies (SRL Inc., Tokyo, Japan).

### Cell line and culture

NHDF-Neo (Normal Human Dermal Fibroblasts - Neonatal) cells were purchased from Lonza (Basel, Switzerland). This cell line was isolated from neonatal foreskin tissue. These cells were cultured in DMEM medium (Nacalai Tesque, Kyoto, Japan) supplemented with 10% FBS, penicillin (100 U/mL), and streptomycin (100 µg/mL) and incubated at 37 °C and 5% CO2. Cells tested negative for mycoplasma contamination.

### Extraction of genomic DNA (gDNA) and PCR analysis

gDNA was extracted from the P3 tumour tissues of the PDX model, the primary tumour tissues, NHDF cells, and normal tissues of BALB/c nude mice using the QIAamp® DNA Mini kit (Qiagen, Hilden, Germany), according to the manufacturer’s protocol. The extracted gDNA was quantified using a NanoDrop 1000 spectrophotometer (Thermo Fisher Scientific, Waltham, MA, USA). Thereafter, 100 ng of gDNA was amplified using Blend Taq® -Plus (TOYOBO, Osaka, JAPAN), and PCR was performed using a 2720 Thermal Cycler (Thermo Fisher Scientific) with the following protocol: 1 cycle at 94 °C for 2 min, followed by 28 cycles at 94 °C for 30 s, 55 °C for 30 s, and 72 °C for 1 min. The PCR products were separated using 1.5% agarose gel electrophoresis with 3 µL of a TrackIt 100-base pair DNA ladder (Invitrogen, Waltham, MA, USA). The amplified fragments were observed by exposing the gel to UV light.

### RNA sequencing

Total RNA was extracted from primary patient and PDX tumour tissues (P3) using the miRNeasy Mini Kit (Qiagen). RNA sequencing was performed by Rarevariant, Inc. (Tokyo, Japan), and the sequencing data were used to quantify the expression level of each gene with Kallisto (http://pachterlab.github.io/kallisto/). The data were summarized using the tximport package (ver. 1.18.0) of R software (ver. 4.1.2) and RStudio (Rstudio, Boston, MA, USA).

Moreover, we used several gene expression omnibus (GEO) datasets for the comparison, including GSE121103, which contains the RNA expression data of clinical samples of epithelial ovarian cancers (EOCs). In addition, GSE192446, GSE190792, GSE131753, GSE43362, and GSE38541 are the gene expression profiles of germ cell tumour cell lines. GSE192446 contains data from our previous study [19]. We used the control-treated cells data of the datasets.

Heatmap and hierarchical clustering analyses were performed using the heatmap.2 function of the gplots package (ver. 3.1.0). The genes with low read counts (< 10 TPM in all samples) were excluded. Moreover, considering several transcripts encode the same gene, only the transcript with the highest expression was used for the heatmap analysis. Differentially expressed genes (DEGs) were identified with an absolute log_2_ fold change > 1. Following comparison of the EOC clinical sample and germ cell tumour cell line data, DEGs in NGC were extracted. Subsequently, Gene Ontology (GO) analysis for the DEGs was performed using an online database, DAVID (https://david.ncifcrf.gov/).

### Statistical analyses

Statistical analyses were performed using GraphPad Prism v.9.4 (GraphPad Software Inc., San Diego, CA, USA). Statistical comparisons between two groups were performed using the Welch’s *t* test. Data are presented as the mean ± standard deviation (SD). *P* < 0.05 was considered statistically significant.

## Results

### Clinical presentation

**Fig. 1:**
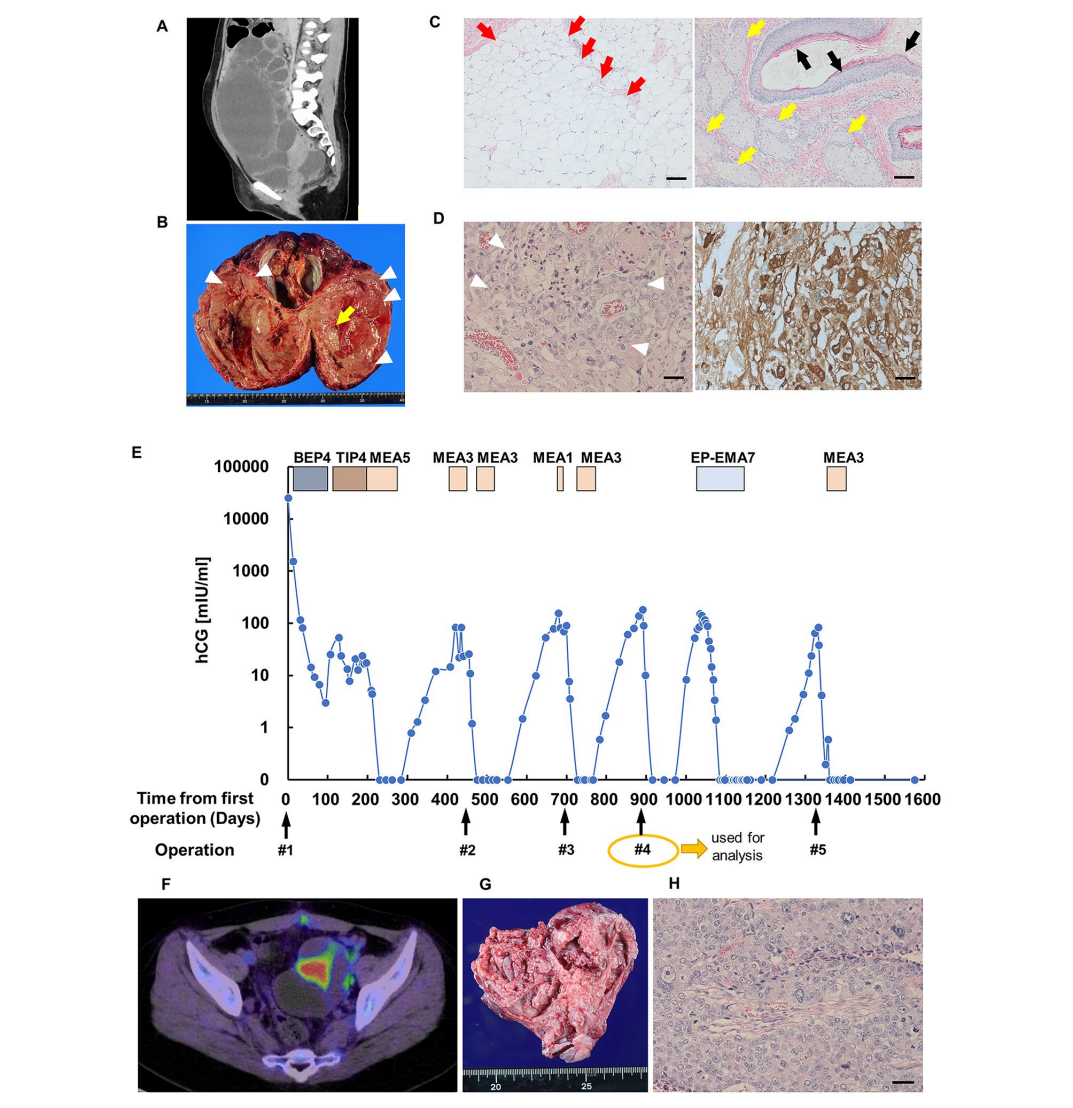
Clinical information of the patient. **(A)** Representative computed tomography (CT) image showing a huge right ovarian tumour in the abdominal cavity. **(B)** Representative image of the resected right ovarian tumour. The arrow indicates mature teratoma portion, and arrow heads indicate choriocarcinoma portion. **(C)** Representative images of H&E-stained tissues of the mature teratoma portion in the right ovarian tumour (first surgery). The arrows indicate mature tissue such as adipose tissue (red), skin (black) and sebaceous glands (yellow). The scale bars represent 100 μm. **(D)** Representative images of H&E staining and immunohistochemistry of the choriocarcinoma portion in the right ovarian tumour (first surgery). The arrowheads indicate cancer tissues that formed destructive tumour lesions with numerous cells containing large trophoblast-like nuclei and also reveal many highly atypical and mitotic figures (left). Immunohistochemistry for hCG showed strong positivity specifically for tumour cells (right). The scale bars represent 50 μm. **(E)** The clinical course of the patient. Details of the regimens used are presented in Additional file 1. The number next to each treatment (e.g., BEP4 and TIP4) indicates the number of chemotherapy cycles. The black arrows indicate surgical time-points. Details of the surgery are as follows: #1, right salpingo-oophorectomy and partial omentectomy; #2, tumour resection in the Douglas; #3, left salpingectomy; #4, left ovarian cystectomy; and #5, left oophorectomy. The sample obtained from the patient during the fourth surgery was used to establish the patient-derived xenograft model. **(F)** Representative image of fluorodeoxyglucose-positron emission tomography indicating accumulation in the left ovary before the fourth surgery. **(G)** Representative image of the left ovarian tumour (fourth surgery). **(H)** Representative image of H&E staining of the left ovarian tumour (fourth surgery). Scale bar, 50 μm

An 18-year-old woman (gravida 0, para 0) was admitted to our hospital with severe abdominal pain and bloating. She had no history of sexual intercourse. Computed tomography (CT) revealed a huge right ovarian tumour (approximately 18 cm × 12 cm × 23 cm) (Fig. 1A), and the serum hCG level measured 25,138.9 mIU/mL. The patient immediately underwent a right salpingo-oophorectomy and partial omentectomy during a first surgery (Fig. 1B). Intraoperative findings revealed that the tumour had spread to the pelvic cavity; hence, the patient was diagnosed with stage IIB cancer. Approximately 2 cm of metastatic lesions were observed in the Douglas fossa and vesicouterine pouch. To achieve complete resection, a combined resection of the rectum and bladder was required. However, due to the high desire for fertility preservation and the expected high chemotherapy sensitivity of malignant germ cell tumours, we determined that resection was not necessary. Pathological examination revealed a mixed germ cell tumour, including choriocarcinoma and a mature teratoma of the ovary (Fig. 1C and D). The patient subsequently received several adjuvant chemotherapy regimens (Fig. 1E and Additional file 1). After 13 cycles of chemotherapy (four cycles of BEP, four cycles of TIP, and five cycles of MEA), the patient achieved remission with negative hCG levels. Moreover, combined positron emission tomography and CT confirmed that there were no measurable lesions, suggesting recurrence or metastases.

However, approximately six months after the completion of chemotherapy, the patient had recurrence in the Douglas fossa. After three cycles of MEA therapy, the recurrent lesion at the Douglas fossa was resected, and the patient achieved macroscopic complete resection (second surgery). Subsequently, she received further chemotherapy (three cycles of MEA), resulting in negative hCG levels. Nevertheless, the patient experienced re-recurrence at the left fallopian tube approximately six months after the last treatment. Due to the strong desire for fertility-sparing, a left salpingectomy (third surgery) was planned after one cycle of MEA therapy. Fortunately, intraoperative findings suggested the tumour was localized to the left fallopian tube, and the pathological examination confirmed the negative margin. As a result, the left ovary was preserved, and three cycles of MEA therapy were administered. However, after approximately another six months, elevated hCG levels were observed, and a recurrent lesion was found in the left ovary. The patient still maintained a strong desire for fertility-sparing; therefore, a left ovarian cystectomy was performed (fourth surgery) without adjuvant chemotherapy, and the tumour was used for the analysis (Fig. 1F–H). A recurrent lesion was found in the left ovary four months after the fourth surgery, and seven cycles of EP-EMA therapy were administered. Approximately six months after the completion of chemotherapy, the left ovary was enlarged again; thus, a left oophorectomy was performed (fifth surgery). Finally, three cycles of MEA therapy were administered, and the patient eventually achieved remission (Fig. 1E). The progression-free period since the end of chemotherapy had been 22 months. Two pathologists confirmed that all recurrent lesions removed during the second and subsequent surgeries exhibited only choriocarcinoma components. Prior to the left oophorectomy, her menstrual cycle had recovered to a normal cycle after the end of chemotherapy.

### The establishment of the PDX model of NGC

A fresh tumour sample was obtained from the patient during the fourth surgery and immediately transplanted into the NSG mouse model. This sample was histologically confirmed to comprise pure choriocarcinoma. The tumours became palpable 42 days after implantation and were transplanted into second-generation mice 76 days after implantation. Subsequently, the PDX model was established through serial passages. The tumour-bearing periods of the P2 and P3 generations were 78 and 61 days, respectively. No metastasis to lungs or other organs was observed in all mouse generations. Moreover, we confirmed that the PDX tumours (P4) were tumorigenic in NSG mice after cryopreservation. The success rate after the transplant was 100%. Furthermore, PDX tumours were engrafted even in BALB/c nude mice. All mice used for PDX establishment did not show weight loss more than 20%.

**Fig. 2:**
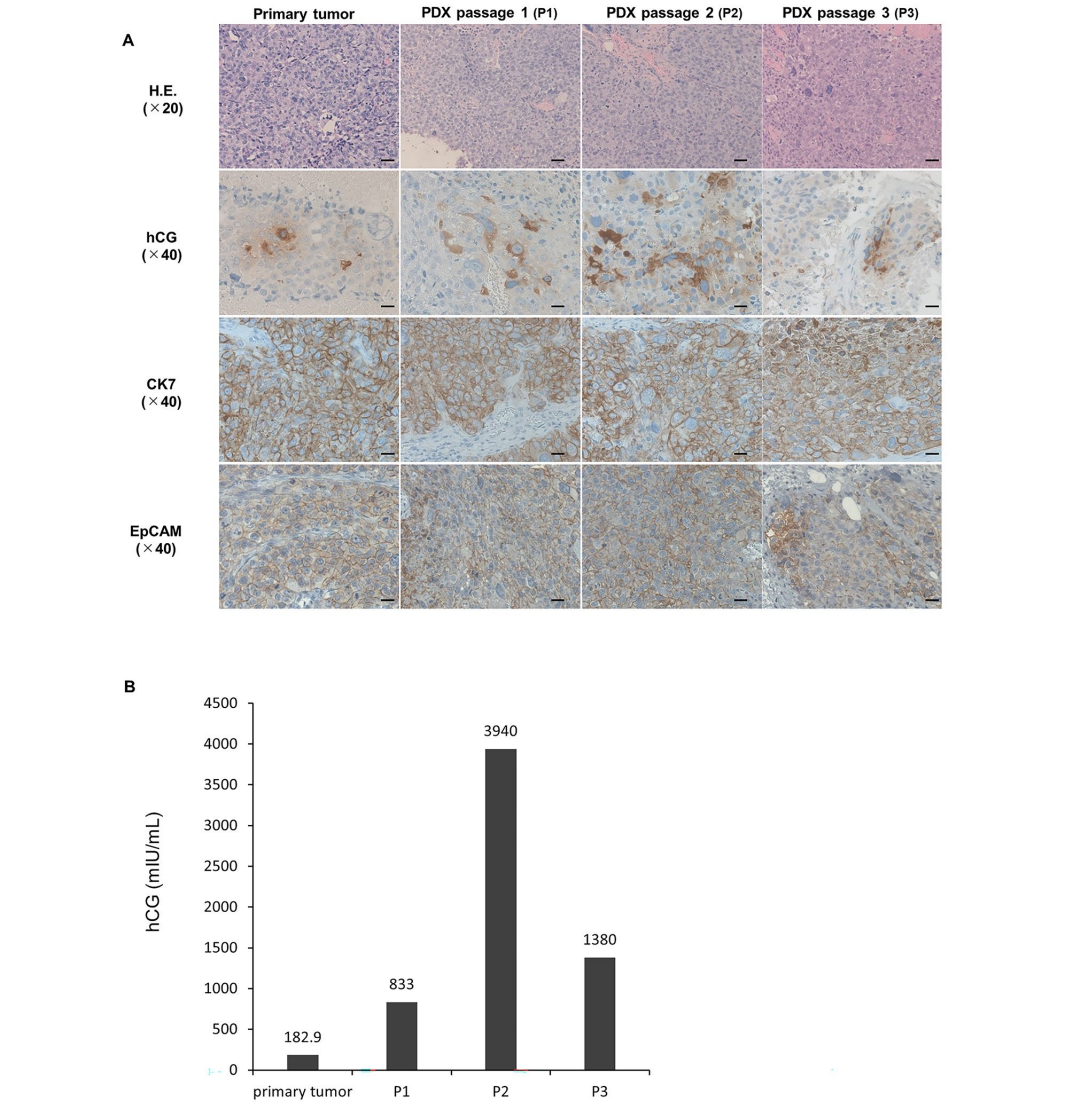
The characteristics of tumours from the patient-derived xenograft (PDX) model. **(A)** Representative images of H&E staining and immunohistochemistry of the primary tumour and PDX models (P1, P2, and P3). The scale bars represent 50 μm (H&E staining) and 20 μm (immunohistochemistry). **(B)** The hCG levels in the patient and the PDX models. The serum hCG level of the patient was measured the day before the fourth surgery. The plasma hCG level of the PDX model was measured when mice were sacrificed (*n* = 1)

Haematoxylin & eosin (H&E) staining showed a substantial proliferation of cells with atypical nuclei of varying sizes in all PDX tumour passages, and tumour cells were arranged in biphasic proliferation of cytotrophoblastic cells rimmed by syncytiotrophoblast cells. The H&E staining findings revealed that primary morphology and tissue architecture were preserved in all PDX tumour passages (Fig. 2A). Additionally, immunohistochemical staining showed that the expression of hCG, CK7, and EpCAM was retained in all passages of PDX tumours. Therefore, the PDX tumours presented the histological characteristics of the original tumour.

Thereafter, we evaluated the serum hCG level of the mice, as this was the biomarker examined in the patient (Fig. 1E). The serum hCG level was remarkably elevated in all passages of the PDX mice (from P1 to P3, Fig. 2B). Therefore, the PDX tumours were considered to secrete hCG, which was consistent with the clinical presentation.

### Further characterization of PDX tumours

**Fig. 3:**
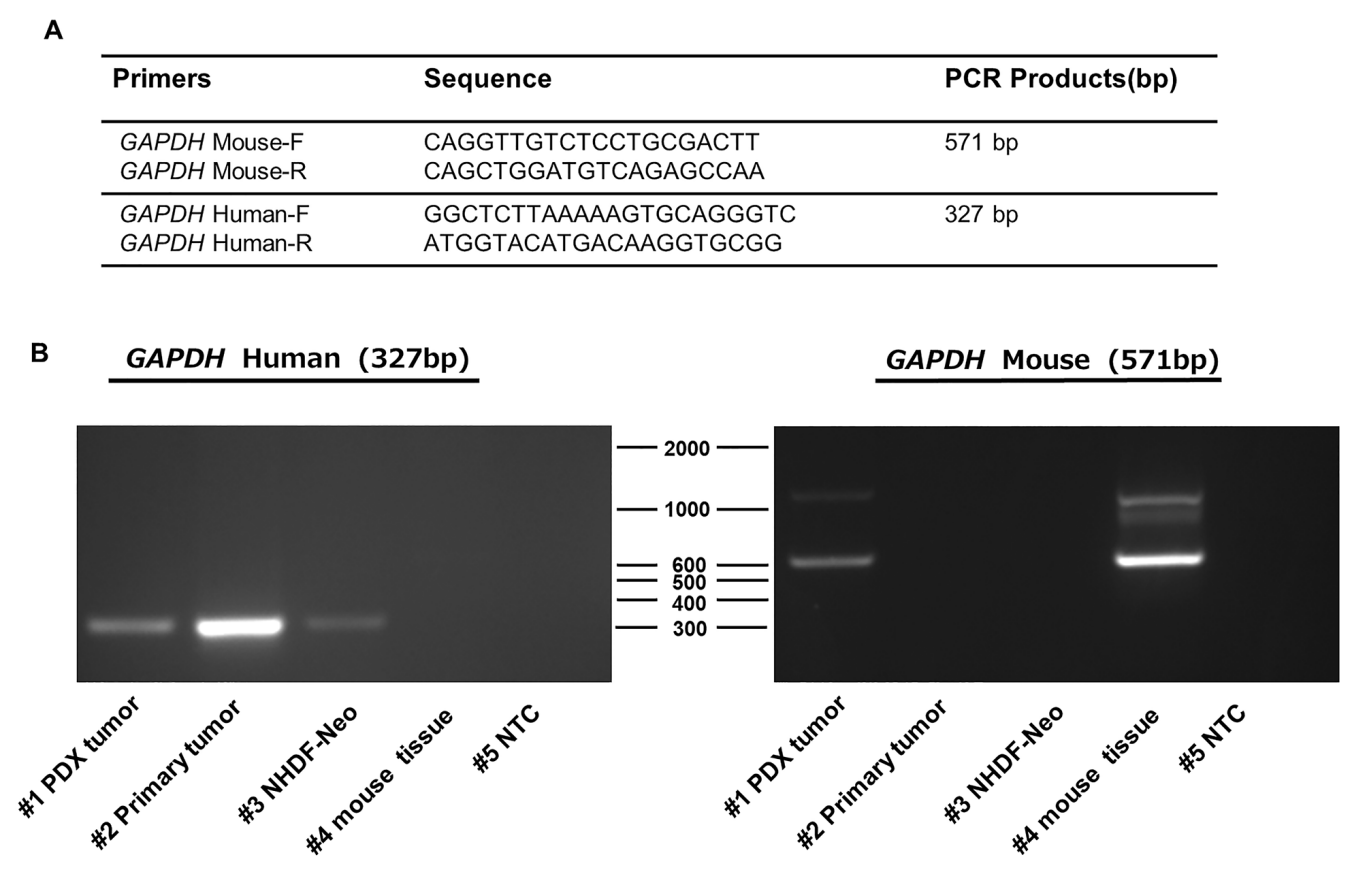
PCR analysis of the patient-derived xenograft (PDX) tumour. **(A)** DNA primers used to detect mouse and human housekeeping genes. **(B)** Electrophoresis images of the PCR products. Lane #1: PDX tumour, Lane #2: Primary tumour, Lane #3: NHDF-Neo, human dermal fibroblasts-neonatal (human genome positive control), Lane #4: mouse tissue (mouse genome positive control), and Lane #5: NTC, no-template control, a template-free control. The full-length gel is presented in Supplementary Information File 1

To validate that the PDX tumours were derived from human tissue, we performed PCR analysis using primers specific for human and mouse *GAPDH*, as shown in Fig. 3A. The primary tumour, NHDF-Neo cells, and normal murine tissues were used as the controls. In the PDX tumour tissue, the expression of both human and mouse *GAPDH* was observed (Fig. 3B). Therefore, the PDX tumour tissue was composed of human cancer cells and normal murine cells.

**Fig. 4:**
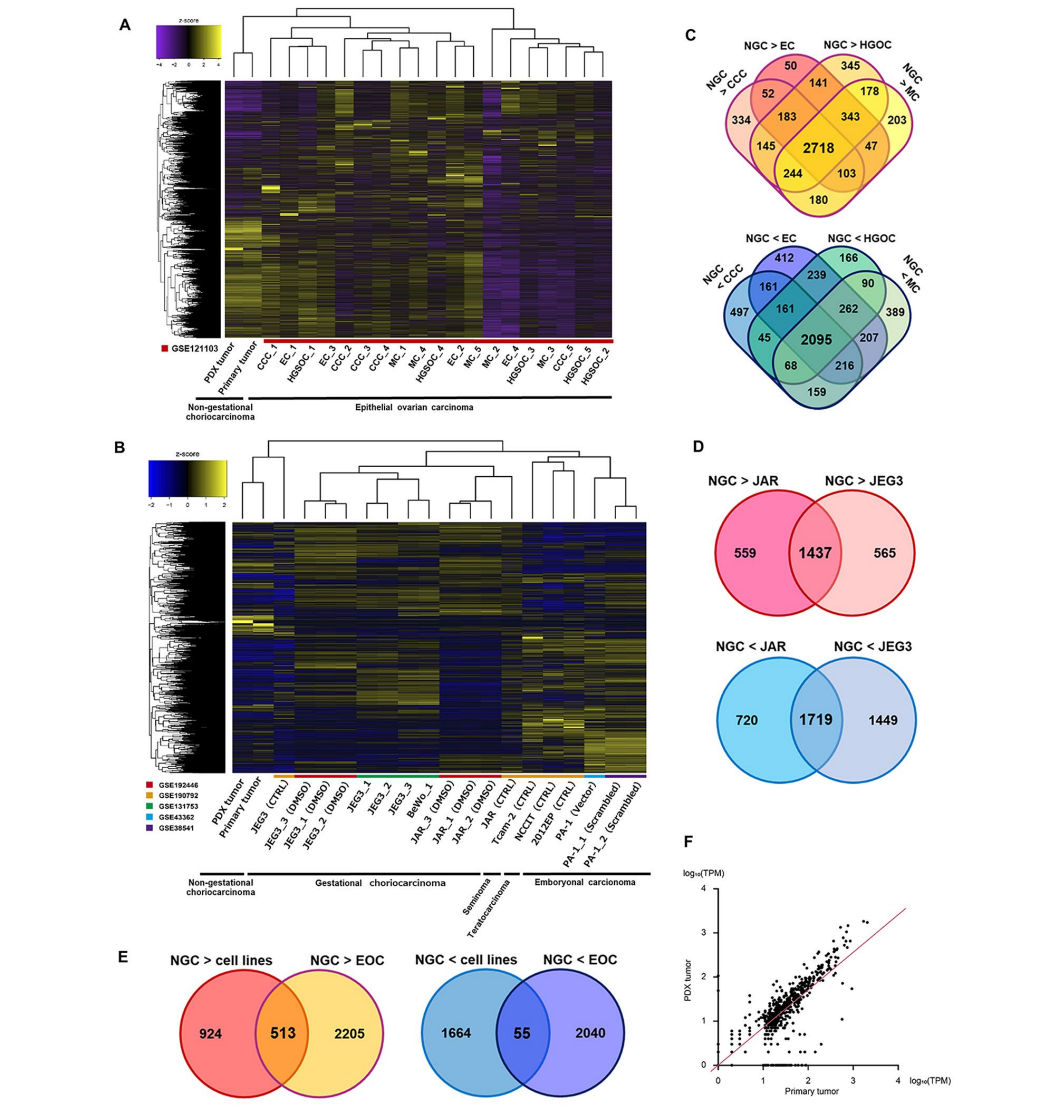
RNA sequencing analysis of the patient-derived xenograft (PDX) tumour. **(A)** Heatmap demonstrating the gene expression of the primary tumour, PDX tumour, and clinical samples of epithelial ovarian cancers (EOC). The GSE121103 dataset was used. CCC, clear cell carcinoma; EC, endometrial carcinoma; HGSC, high grade serous carcinoma; MC, mucinous carcinoma **(B)** Heatmap demonstrating the gene expression of primary tumour, PDX tumour, and cell lines. The GSE192446, GSE190792, GSE131753, GSE43362, and GSE38541 datasets were used. **(C)** Venn diagrams showing differentially expressed genes (DEGs) in non-gestational choriocarcinoma (NGC) compared to each histology of EOC. **(D)** Venn diagrams showing DEGs in NGC compared to those in gestational choriocarcinoma cell lines (JAR and JEG3 in the GSE192446 dataset). **(E)** Venn diagrams showing the common DEGs in NGC compared to those in clinical samples of EOC and gestational choriocarcinoma cell lines. **(F)** The correlation of the 568 DEGs (513 upregulated and 55 downregulated genes) between the primary and PDX tumours

Thereafter, we assessed gene expression in the PDX tumour (P3) using RNA-seq and multiple GEO datasets. Heatmap analysis showed that the gene expression profiles of PDX were more similar to those of the primary tumour than those of EOC tissue. (Fig. 4A). Similarly, when compared to the cell lines of GC and germ cell tumour, the PDX tumour showed gene expression patterns similar to those of the primary tumour (Fig. 4B). Thus, the PDX and primary tumours had unique gene expression profiles. Next, we performed Venn diagram analysis to extract the unique genes of NGC. Compared with each histology of EOC, 2,718 and 2,095 genes were commonly upregulated and downregulated, respectively, in NGC (Fig. 4C). Moreover, compared with the GC cell lines (JAR and JEG3) of GSE192446, 1,437 and 1,719 genes were commonly upregulated and downregulated, respectively, in NGC (Fig. 4D). We extracted 568 DEGs of NGC (513 upregulated and 55 downregulated; Fig. 4E and Additional file 2). Finally, we confirmed that the expression of the 568 DEGs was highly correlated between primary and PDX tumours (correlation coefficient = 0.873, *P* < 0.001, Fig. 4F). Moreover, the GO term analysis suggested that the 568 DEGs might be associated with the angiogenesis and chemotaxis of immune cells (Additional File 3). Further research is required to elucidate the significance of the DEGs in NGC in more detail.

### Drug response of PDX models

**Fig. 5:**
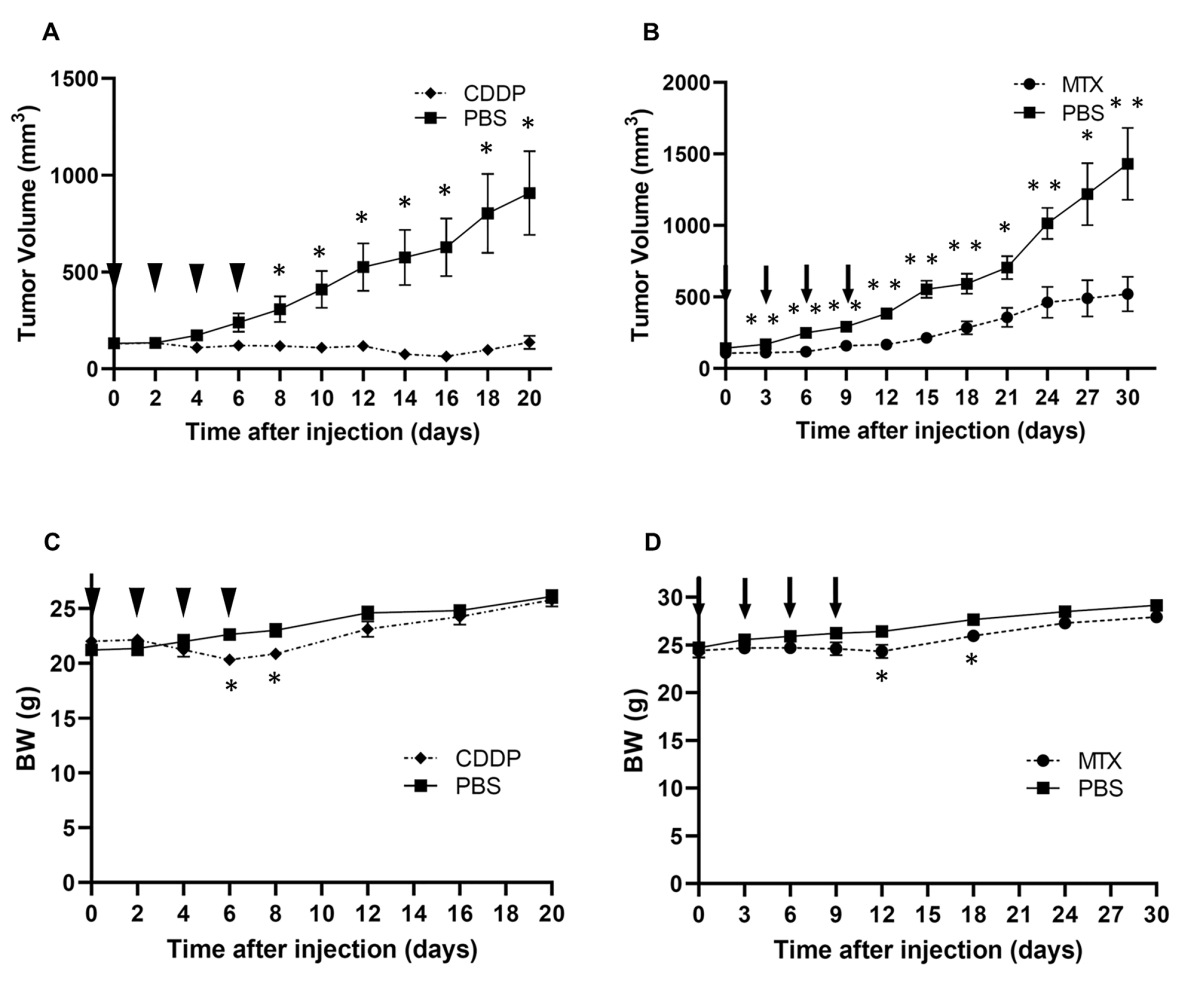
Effect of chemotherapy in the patient-derived xenograft (PDX) model. **(A)** Tumour volume of cisplatin (CDDP)-treated nude mice (n = 5). **(B)** Tumour volume of methotrexate (MTX)-treated nude mice (n = 5). **(C)** Body weight of the CDDP-treated nude mice. **(D)** Body weight of MTX-treated nude mice. The arrowheads indicate the administration of CDDP (2 mg/kg body weight) or PBS, and the arrows indicate the administration of MTX (50 mg/kg body weight) or PBS. The error bars represent the mean ± standard deviation. The tumour volume and weight were compared using Welch’s *t-*test, *P < 0.05 and **P < 0.01

Finally, to evaluate the responsiveness of the PDX model to CDDP and MTX (which are clinical treatments used for choriocarcinoma), we analysed their effects on tumorigenicity in nude mice. We found that treatment with CDDP significantly reduced the tumour volume compared to that of the control (P < 0.05, Fig. 5A). Similarly, tumour volume was significantly reduced when treated with MTX compared to that of the control (P < 0.01, Fig. 5B). In addition, both drug treatment groups showed temporary weight loss, with statistical significance, during treatment (P < 0.05, Fig. 5C,D). Therefore, the PDX model could reproduce patient-specific responses to chemotherapeutic drugs.

## Discussion

In the present study, a PDX model from a patient with NGC was established successfully. A previous report has demonstrated a mini-PDX model for NGC [20]. The PDX model and mini-PDX model are different techniques. For example, in the mini-PDX model, the duration of treatment is limited to up to 7 days, and intercellular the structure is disrupted during the establishment phase. Therefore, our PDX model certainly has advantages and would facilitate further NGC research.

The clinical course showed that the patient had refractory cancer, which repeatedly relapsed despite several chemotherapeutic regimens and multiple surgeries. The aggressiveness of the tumour may be associated with our successful establishment of a PDX model. In a previous study, patients whose cancers failed to engraft into nude mice had an 81% reduction in the risk of death from pancreatic cancer [21]. In general, PDX engraftment rates are between 25% and 75%, depending on the tumour type and engraftment techniques applied [22, 23]. Several factors are believed to contribute to this wide variation range, including the quality of patient tumour tissues used for transplantation, location of the implantation site, and type of immune-deficient mouse. Regarding the patient tumour tissue quality, as fresh and sufficient tumour volume is required for transplantation, success rates may be affected by the time interval from tissue collection to transplantation, which affects the extent of tumour necrosis [24]. With respect to the location of implantation in SCID mice, a previous report on ovarian cancer showed that the PDX engraftment success rates for subcutaneous, mammary fat pad, intraperitoneal, and sub-renal capsules have been reported as 85.3%, 63.6%, 22.2%, and 8.3%, respectively [14]. In terms of immune-deficient mouse type, an improved engraftment rate can be achieved by implantation into SCID or NOD-SCID-IL2R mice rather than BALB/c nude mice [25]. However, the high cost of SCID mice makes them less versatile. In this study, given the rarity of NGC tumours, we used NOD-SCID-IL2 to ensure an efficient engraftment rate and successfully established a PDX model. The subcutaneous implantation location was chosen because of the ease of the procedure, and the time between implantation and development of the palpable xenograft tumour in first-generation mice was approximately 6 weeks. This short period may reflect the aggressiveness of the primary tumour, in addition to differences in tumour type.

When comparing histological and gene expression profiles, we found a high degree of similarity between the PDX model and primary tumour. Similar to GC, NGC reveals the abnormal proliferation of atypical trophoblasts histologically and presents a two-cell pattern organized into two types of trophoblastic cells: syncytiotrophoblastic and cytotrophoblastic. Trimorphic proliferation was observed in certain cases, including in intermediate trophoblasts. These structures are mixed and proliferate in full or sheet-like forms, infiltrate, and proliferate in the surrounding tissues and blood vessels. They are often associated with haemorrhage and necrosis. In addition, the chorionic villi are absent [26, 27]. H&E staining of our PDX model showed the histological characteristics of choriocarcinoma with mixed syncytiotrophoblastic and cytotrophoblastic cells having proliferated to fullness in all generations from the first to the third. Haemorrhage and necrosis were also observed. In addition to its morphological features, hCG is a noteworthy tumour marker for NGC [26]. As hCG is secreted by syncytiotrophoblast cells, hCG positivity in immunohistochemistry is auxiliary to the diagnosis of NGC. The expression of CK7 has been specifically observed in trophoblast and trophoblast-derived tumour cells and is regarded as the best marker for trophoblasts [28]. EpCAM has also been reported to be positive in choriocarcinoma [29]. In our PDX model, such expression patterns were consistent with those of the primary tumour, suggesting that the characteristics of NGC were maintained.

Furthermore, as hCG is secreted into the blood, serum hCG levels are extremely useful biomarkers for periodic monitoring to evaluate a therapeutic response [30]. Our PDX model also showed elevated plasma hCG levels, which might reflect disease activity. However, there was no correlation between tumour volume and serum hCG levels. In addition, serum hCG was not measured during periods when the tumour was nonpalpable.

Furthermore, the heatmap analysis of RNA-seq revealed the similarity of gene expression profiles between PDX and primary tumours. These results are consistent with previous studies comparing PDX and corresponding patient tumours in breast and colon cancer [11, 31].

In PDX, human lymphoma and mouse tumours can develop spontaneously [32–34]. Therefore, the presence of human gene was confirmed using PCR. PCR verified the expression of a human gene in the PDX tumour. Furthermore, mouse genes were detected in PDX tumours, suggesting that stromal cells were derived from the host.

Given that the response to drugs is important for evaluating the clinical predictive value of any PDX model, we validated whether the results of drug intervention using our PDX model were comparable to the clinical outcomes of NGC. The most commonly used chemotherapy for NGC is a platinum-based regimen, including BEP and TIP therapies, which is based on a treatment for MGCT [2, 6]. Moreover, considering histologic similarities to GC, MTX-based regimens, such as MEA and EP/EMA therapies, may be effective for NGC [2]. We demonstrated that both CDDP and MTX exhibit the in vivo anti-cancer effect, and these results might reflect the clinical course of the patient who achieved remission after seven cycles of CDDP- and MTX-based chemotherapy. Therefore, our PDX model can be useful for predicting drug responses and would be a powerful tool for drug discovery research in NGC.

This study had some limitations. First, only one PDX model was established. However, NGC is a very rare cancer, and it occurs in only three cases in our institution in the past 10 years. Therefore, it needs too much time to establish several PDX models, and we have to keep trying to establish several PDX models of NGC. Second, the RNA-seq data used to evaluate gene expression may not be optimal. Hence, the subsequent pathway analysis might have limited significance. Third, the genetic characteristics of NGC were not assessed. Therefore, to further research about the molecular background of NGC using our PDX model, whole genome sequencing (WGS) is essential for evaluating the genetic similarity between the PDX model established and the original tumour. Fourth, the sensitivity to CDDP and MTX was evaluated. In the present study, to evaluate whether the NGC-derived PDX model reflects the properties of both MGCT and GCN, CDDP was selected as a key drug for MGCT and MTX, as an important drug for GTN. However, to further clarify the drug sensitivity of the PDX model, the effects of other drugs, such as etoposide and bleomycin, should be assessed in future.

## Conclusions

A PDX model of NGC was established and a high degree of similarity between the PDX and primary tumours was demonstrated. The results suggested that the PDX model represents the histopathological and gene expression characteristics of primary tumours. Therefore, the model could provide a promising platform for novel therapeutic strategies for NGC. Further studies are required to improve the prognosis of patients with NGC.

### Electronic supplementary material

Below is the link to the electronic supplementary material.

Supplementary Material 1

Supplementary Material 2

Supplementary Material 3

Supplementary Material 4

## Data Availability

The study’s GEO database (GSE230352) can be downloaded from https://www.ncbi.nlm.nih.gov/gds/.

## List of abbreviations

CDDP: Cisplatin
EOC: Epithelial ovarian cancer
GC: Gestational choriocarcinoma
gDNA: Genomic DNA
GTN: Gestational trophoblastic neoplasia
GEO: Gene expression omnibus
hCG: Human chorionic gonadotropin
MGCT: Malignant germ cell tumours
MTX: Methotrexate
NGC: Non-gestational choriocarcinoma
PDX: Patient-derived xenograft
