# Supplementary material for: Establishment and characterization of a non-gestational choriocarcinoma patient-derived xenograft model
Source: BMC Cancer. 2023 Nov 13;23:1103. doi: 10.1186/s12885-023-11626-3 (PMC10642054; doi:10.1186/s12885-023-11626-3)
Supplement: Supplementary file 1 — Supplementary Material 1 [file 12885_2023_11626_MOESM1_ESM.docx]

| BEP | Day1-5  　Etoposide  　Cisplatin  Day2,9,16  　Bleomycin | 100 mg/m^2^  20 mg/m^2^  30 mg | iv. infusion over 2 h  iv. infusion over 2 h  iv. infusion over 15 m |
| --- | --- | --- | --- |
| TIP | Day1  　Paclitaxel  Day2-6  　Ifosfamide  　Cisplatin | 175 mg/m^2^  1.2 g/m^2^  20 mg/m^2^ | iv. infusion over 3 h  iv. infusion over 2 h  iv. infusion over 2 h |
| MEA | Day1  　Methotrexate  Methotrexate  　Etoposide  　Actinomycin D  Day2-4  　Etoposide  　Actinomycin D  　Folinic acid | 150 mg  300 mg  100 mg  0.5 mg  100 mg  0.5 mg  15mg | iv. bolus just prior to the methotrexate infusion  iv. infusion over 4 h  iv. infusion over 2 h  iv. bolus  iv. infusion over 2 h  iv. bolus  iv. every 12 h×3 starting 24 h after commencement  of methotrexate |
| EP-EMA | Day2  　Etoposide  　Cisplatin  Day9  　Etoposide  　Actinomycin D  　Methotrexate  Day10,11  　Folinic acid | 150 mg/m^2^  75 mg/m^2^  100 mg/m^2^  0.5 mg  300 mg/m^2^  15 mg | iv. infusion over 2 h  iv. infusion over 2 h  iv. infusion over 2 h  iv. bolus  iv. infusion over 12 h  iv. every 12 h×4 starting 24 h after commencement  of methotrexate |

Additional file 1 Protocol of chemotherapies

Table listing the protocol of chemotherapies administered to the patient over the clinical course.

BEP, etoposide, cisplatin and bleomycin; TIP, paclitaxel, ifosfamide and cisplatin; MEA, methotrexate, etoposide and actinomycin D; EP-EMA, etoposide, cisplatin, etoposide, actinomycin D, methotrexate and folinic acid; iv, intravenous
