# Supplementary figures and images for: Establishment and characterization of a non-gestational choriocarcinoma patient-derived xenograft model
Source: BMC Cancer. 2023 Nov 13;23:1103. doi: 10.1186/s12885-023-11626-3 (PMC10642054; doi:10.1186/s12885-023-11626-3)

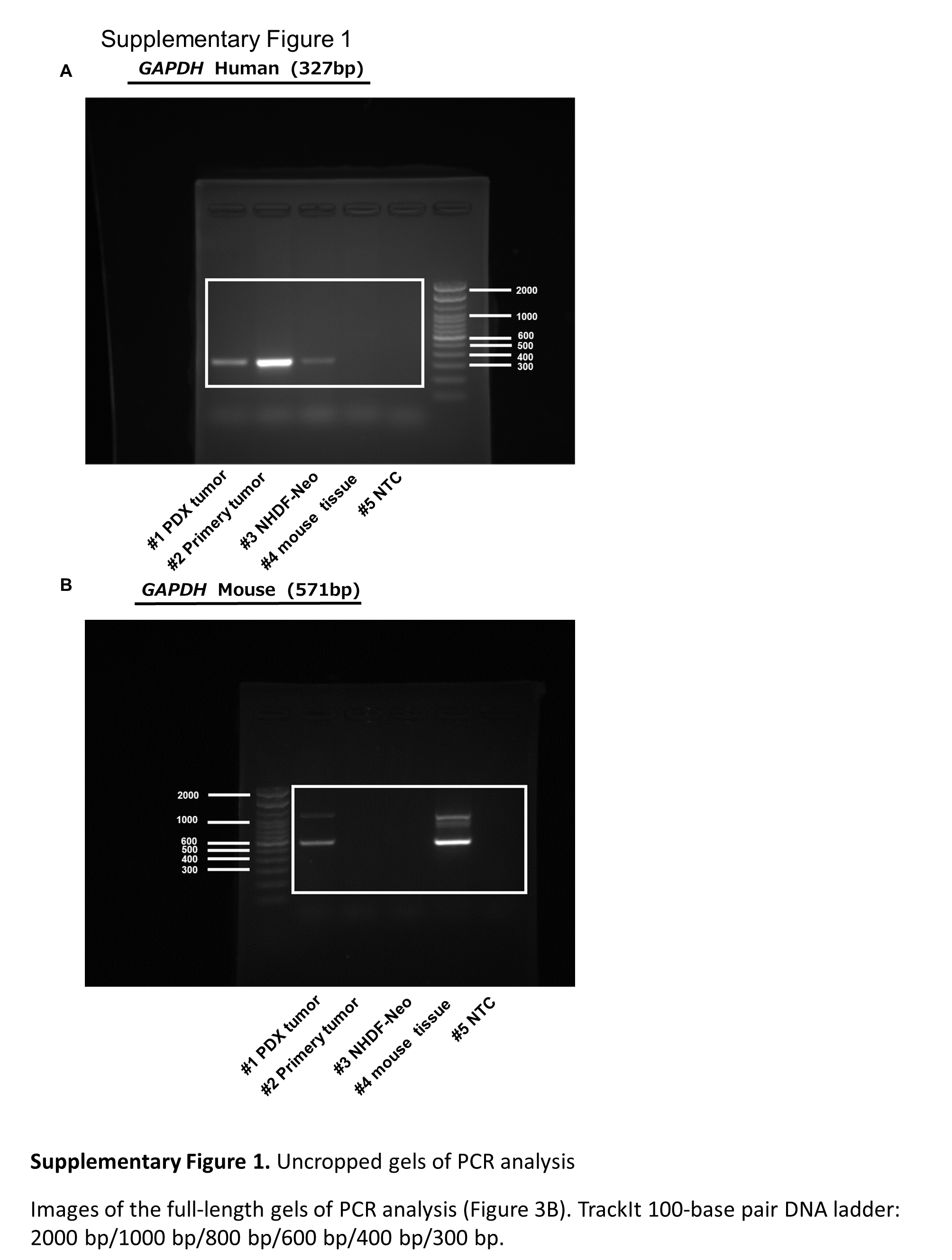

Supplement: Supplementary file 4 — Supplementary Material 4 [file 12885_2023_11626_MOESM4_ESM.tif]
